# Association Between Strabismus and Risk of Open-Angle Glaucoma: A Multicenter Retrospective Cohort Study

**DOI:** 10.3390/jcm15155823

**Published:** 2026-07-25

**Authors:** Chen-Ya Hung, Shu-Han Chuang, Bing-Hua Lin, Yu-Pin Chen, Yi-Jie Kuo, Cheng-Hsien Chang, Lien-Chen Wu, Wei-Yang Lu

**Affiliations:** 1 School of Medicine, College of Medicine, Taipei Medical University, Taipei 11031, Taiwan; 2 Department of Ophthalmology, Changhua Christian Hospital, Changhua 50006, Taiwan; 3 Division of General Practice, Department of Medical Education, Changhua Christian Hospital, Changhua 50006, Taiwan; 4 Department of Orthopedics, Wan Fang Hospital, Taipei Medical University, Taipei 11031, Taiwan; 5 Department of Orthopedics, School of Medicine, College of Medicine, Taipei Medical University, Taipei 11031, Taiwan; 6 Department of Post-Baccalaureate Medicine, College of Medicine, National Chung Hsing University, Taichung 40227, Taiwan; 7 Graduate Institute of Biomedical Materials and Tissue Engineering, College of Biomedical Engineering, Taipei Medical University, Taipei 11031, Taiwan; 8 Department of Optometry, Chung Shan Medical University, Taichung 40242, Taiwan

**Keywords:** strabismus, glaucoma, open-angle, ocular hypertension, antiglaucoma agents, electronic health records, cohort studies

## Abstract

**Background/Objectives:** To evaluate the longitudinal association between strabismus and the subsequent risk of developing open-angle glaucoma (OAG), ocular hypertension (OHT), and antiglaucoma agent (AGA) initiation or OAG interventions in patients aged 65 years and older. **Methods:** We conducted a multicenter retrospective cohort study using the TriNetX US Collaborative Network from 1 January 2003 to 31 December 2022. Patients aged 65 years or older with an ophthalmic encounter and a first recorded diagnosis of paralytic or other strabismus were compared with patients without strabismus. Individuals with prior glaucoma, AGA use, or glaucoma procedures before or on the index date were excluded. One-to-one propensity score matching was performed for demographics, ophthalmic conditions, systemic comorbidities, healthcare utilization, and medication history. Cox models and Kaplan–Meier analyses were used to estimate 3-year risks; 1-year lag-time, follow-up duration, subtype, stratified, and control-outcome analyses were performed. **Results:** After matching, 20,894 patients were included in each cohort. Strabismus was not associated with a higher 3-year risk of OAG (hazard ratio (HR) = 0.833; 95% confidence interval (CI) = [0.683–1.017]), OHT (HR = 0.923; 95% CI = [0.825–1.031]), or OAG interventions (HR = 0.851; 95% CI = [0.691–1.048]). AGA initiation was lower in the strabismus cohort (HR = 0.629; 95% CI = [0.574–0.690]). Findings were generally consistent across lag-time and subtype analyses. **Conclusions:** First-recorded strabismus was not associated with an increased recorded risk of OAG, OHT, or OAG interventions among adults aged 65 years or older. Standard glaucoma screening should remain individualized according to established risk factors and examination findings.

## 1. Introduction

Strabismus is a disorder of horizontal, vertical, or torsional ocular misalignment. Although it is most often discussed as a pediatric condition, strabismus in older adults has distinct diagnostic and therapeutic implications. In this age group, it may arise from trauma, prior ocular surgery, thyroid dysfunction, cranial nerve palsies, or neurologic disease [1,2,3]. Acute or newly recognized strabismus can therefore function as a sentinel sign of serious neurologic or vascular pathology. Epidemiologic data also suggest that the annual incidence of adult-onset strabismus increases with age [1]. Beyond diagnostic significance, strabismus can substantially impair quality of life. Many older adults present with diplopia, and the resulting visual disturbance may interfere with reading, driving, mobility, and other daily activities [4,5]. Psychosocial burden is also considerable, with vision-related quality-of-life impairment reported to be comparable to or worse than that seen in major blinding eye diseases [6]. Surgical correction is often pursued to restore alignment, relieve symptoms, and improve function [7,8].

Strabismus and glaucoma are both common in older adults, raising the possibility of a clinically relevant association. Experimental imaging, physiological, and biomechanical studies suggest that horizontal ocular duction and extraocular muscle contraction may transmit traction through the optic nerve sheath and posterior globe, resulting in globe retraction, deformation of the optic nerve head and peripapillary tissues, and changes in regional tissue strain [9,10,11,12]. These tractional forces may also alter optic nerve head perfusion and episcleral venous outflow, thereby modifying the mechanical and vascular environment of the optic nerve [11,12]. These effects may be particularly relevant in older adults because age-related connective tissue remodeling, reduced tissue compliance, and increased scleral stiffness could limit the capacity of the posterior globe to accommodate repetitive deformation [13,14,15]. Eye-movement abnormalities reported in patients with glaucoma further support a possible relationship between ocular motility and glaucoma susceptibility [16]. Nevertheless, these findings primarily provide biomechanical and physiological plausibility and do not demonstrate that strabismus independently causes glaucoma in clinical populations.

In contrast to the primarily experimental evidence described above, the available clinical evidence is limited and has largely been derived from cross-sectional studies. Previous studies have reported associations between strabismus and open-angle glaucoma (OAG). One study reported greater odds of primary open-angle glaucoma (POAG) and normal-tension glaucoma (NTG) among individuals with strabismus [17], while another found a markedly higher prevalence of POAG among individuals with esodeviation than among those without esodeviation [10].

However, these cross-sectional findings cannot establish temporal sequence or determine whether the observed associations reflect a direct biomechanical effect, shared risk factors, or differential ophthalmic surveillance. Previous clinical studies have primarily focused on established glaucoma, often overlooking the pre-glaucomatous phase or ocular hypertension (OHT), which may represent an earlier glaucoma-related clinical phenotype. To address these knowledge gaps, we used the TriNetX database to conduct a longitudinal analysis. We aimed to evaluate not only incident OAG, but also earlier or related outcomes, including OHT, antiglaucoma agent (AGA) initiation, and glaucoma-related laser or surgical interventions. Based on the proposed biomechanical mechanisms and previous cross-sectional evidence, we hypothesized that older adults with strabismus would have a higher subsequent hazard of OAG and OHT than matched adults without strabismus.

## 2. Materials and Methods

### 2.1. Data Source and Study Design

This multi-institutional, retrospective cohort study utilized the TriNetX analytics platform (TriNetX, LLC, Cambridge, MA, USA; https://trinetx.com/, accessed on 24 February 2026). TriNetX is a global federated health research network that provides real-time access to electronic medical records—including diagnoses, procedures, medications, laboratory values, and genomic information—across large healthcare organizations (HCOs). This study primarily accessed the US Collaborative Network within TriNetX, which includes data from 71 HCOs.

TriNetX is compliant with the Health Insurance Portability and Accountability Act (HIPAA) and is certified to the ISO/IEC 27001:2022 standard. TriNetX maintains an Information Security Management System to protect the healthcare data to which it has access and to meet the requirements of the HIPAA Security Rule [18,19]. Data displayed on the TriNetX platform in aggregate form contain only de-identified information in accordance with the de-identification standard defined in 45 CFR § 164.514(a) of the HIPAA Privacy Rule. The de-identification process is attested to through a formal determination by a qualified expert, as defined in 45 CFR § 164.514(b)(1) of the HIPAA Privacy Rule. According to TriNetX, this expert determination was refreshed in December 2020. Only de-identified aggregate counts and statistical summaries were exported from the platform.

The study was approved by the Institutional Review Board of Changhua Christian Hospital (approval number 250612). This research followed the principles of the Declaration of Helsinki and was conducted in accordance with the Strengthening the Reporting of Observational Studies in Epidemiology (STROBE) reporting guidelines.

### 2.2. Study Population and Cohort Definition

**Figure 1:**
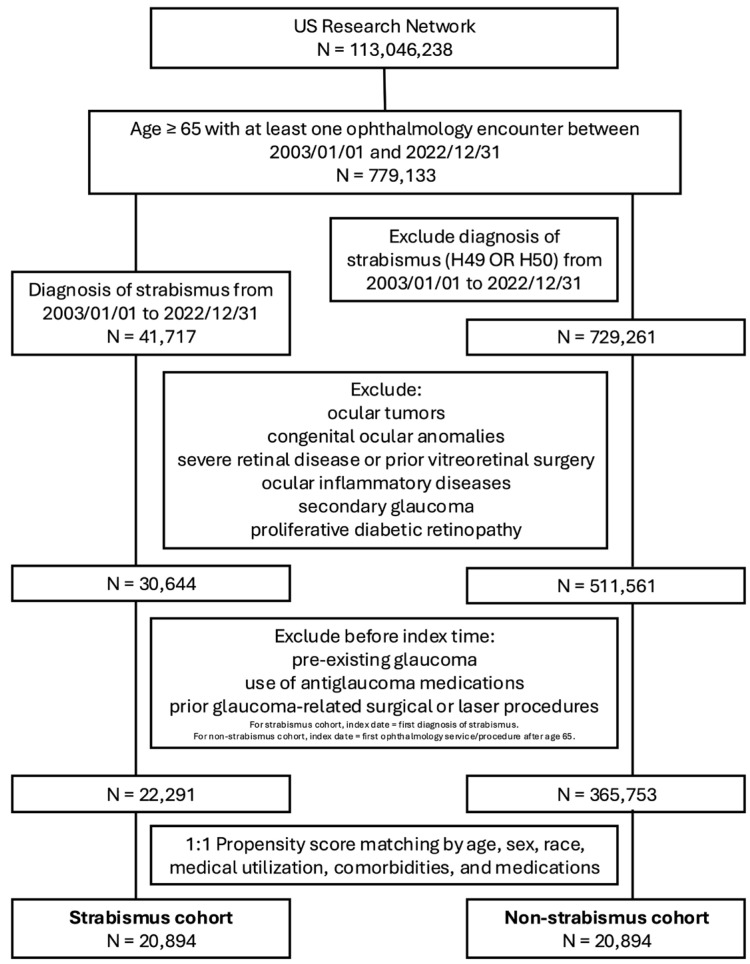
Flowchart of patient selection.

The cohort construction process is illustrated in Figure 1. The study compared glaucoma-related outcomes between two cohorts: Strabismus and non-strabismus, spanning from 1 January 2003 to 31 December 2022.

In the TriNetX database, the strabismus cohort comprised individuals aged 65 years or older with at least one ophthalmic encounter and a first recorded diagnosis of paralytic strabismus or other strabismus; the non-strabismus cohort consisted of patients aged 65 years or older who had at least one ophthalmologic encounter and no recorded diagnosis of strabismus. Individuals with histories of malignant or benign neoplasms of the eye, retinal detachment repair, vitrectomy, eye injuries, or congenital malformations of the eye were excluded to minimize confounding variables. To ensure the accuracy of incident outcome assessments, exclusion criteria included a history of glaucoma, prior use of antiglaucoma medications, or glaucoma-related procedures (e.g., goniotomy, trabeculotomy, minimally invasive glaucoma surgery, or laser trabeculoplasty) on or before the index date. Detailed coding algorithms for cohort identification are provided in Tables S1 and S2.

### 2.3. Index Date and Follow-Up Period

The index event was defined as the date a patient first met the inclusion criteria for their respective cohort. For the strabismus cohort, the index date was defined as the date of the first recorded diagnosis of paralytic or other strabismus in the database, representing the first recorded diagnosis within TriNetX. For the non-strabismus cohort, the index date was assigned as the date of the first ophthalmologic encounter after age 65 that satisfied all other inclusion and exclusion criteria. The follow-up window for analyzing outcomes was set from 1 day to 3 years after the index event. Patients contributed follow-up from 1 day after the index date until outcome occurrence, the end of the specified follow-up window, or the last available record within the TriNetX dataset, according to platform time-to-event settings.

### 2.4. Outcomes

The primary outcomes of interest included the development of OAG, including primary, unspecified, or low-tension glaucoma, and OHT, including borderline findings or high-risk open angles. The secondary outcomes were AGA initiation, including prostaglandin analogs, beta-blockers, alpha agonists, carbonic anhydrase inhibitors, miotics, antiglaucoma combination medications, and netarsudil, and OAG interventions, encompassing surgical or laser interventions such as trabeculectomy, aqueous shunt procedures, and ciliary body destruction. An outcome was considered present upon the first qualifying ICD-10-CM diagnosis code recorded during follow-up. Repeated diagnostic codes, standardized ophthalmic examination findings, and central chart adjudication were not required by the TriNetX outcome algorithm. Detailed coding algorithms for outcomes are provided in Table S3.

### 2.5. Positive and Negative Control Outcomes

To assess the internal validity of the study design, diplopia was selected as the positive control because it is a well-recognized clinical manifestation associated with strabismus. Umbilical hernia was selected as the negative control because it has no plausible biological or clinical relationship with strabismus, ocular biomechanics, or glaucoma-related outcomes, while remaining a routinely recorded diagnosis within electronic health records (Table S3).

### 2.6. Propensity Score Matching and Covariates

To account for baseline differences between the cohorts, 1:1 propensity score matching (PSM) was performed using the built-in matching algorithm of the TriNetX platform. Propensity scores were estimated using logistic regression based on prespecified baseline covariates. Patients were matched on demographics (age, sex, race, and ethnicity), family history of glaucoma, ophthalmic conditions (myopia, hypermetropia, astigmatism, degenerative myopia, age-related cataract, other cataract, disorders of the lacrimal system, corneal scars or opacities, corneal disorders, and orbital disorders), healthcare utilization (inpatient admissions, outpatient visits, and ophthalmology services), comorbidities (metabolic, cardiovascular, renal, hepatic, neurologic, respiratory, rheumatologic, and behavioral factors), and medication history (corticosteroids, beta-blockers, calcium channel blockers, and renin–angiotensin system agents). Greedy nearest-neighbor matching without replacement was performed using a caliper of 0.10 pooled standard deviations of the propensity score. The cohorts were randomly shuffled before matching. Covariate balance was assessed using absolute standardized mean differences (SMDs), with values below 0.10 considered indicative of adequate balance.

Because the analyses were conducted within TriNetX using de-identified electronic health record data, no individual-level imputation was performed. Analyses were based on available recorded data, and unknown or unrecorded demographic categories were retained as separate categories when provided by the platform.

### 2.7. Subgroup, Stratified, and Sensitivity Analyses

To investigate whether the association between strabismus and glaucoma risk varied across different anatomical and clinical presentations, subgroup analyses were performed based on specific International Classification of Diseases, Tenth Revision, Clinical Modification (ICD-10-CM) classifications. The prespecified subtypes were paralytic strabismus (H49), esotropia (H50.0), exotropia (H50.1), and vertical strabismus (H50.2) [17]. For each subgroup, we evaluated the risk of 3-year risk of OAG, OHT, AGA initiation, and the requirement for OAG interventions.

To ensure the robustness of the associations and to mitigate potential protopathic bias, a 1-year lag-time analysis was implemented. Patients diagnosed with the outcomes of interest within the first 12 months following the index strabismus diagnosis were excluded, and the observation window was shifted to the period between year 1 and year 3. We also examined the impact of varying follow-up durations by comparing short-term (1-day to 1-year) and long-term (1-day to 5-year) outcomes. These temporal evaluations aimed to determine whether the observed associations remained consistent across different follow-up periods or were concentrated shortly after the initial ophthalmic encounter.

To explore the potential modification of risk by patient factors and systemic health status, stratification analyses were conducted. Patients were stratified by age as 65–74 years and ≥75 years, race strata including White, Black/African American, and Asian patients, and comorbidity strata including hypertension, hyperlipidemia, diabetes mellitus, nicotine dependence, ischemic heart disease, sleep disorders, chronic lower respiratory disease, and age-related cataract.

### 2.8. Statistical Analysis

Baseline covariate balance between the cohorts was evaluated using standardized mean differences (SMD), with an SMD of less than 0.1 indicating adequate balance. The risks of OAG and related outcomes were estimated using Cox proportional hazards regression models implemented within the TriNetX platform according to its default analytical settings, and results were reported as hazard ratios (HRs) with corresponding 95% CIs. Cumulative incidence was illustrated using Kaplan–Meier analysis, and differences between cohorts were assessed with the log-rank test. The proportional hazards assumption was formally assessed for each Cox regression model using the proportionality test provided by the TriNetX platform. A proportionality-test *p* value < 0.05 was considered evidence of potential violation of the proportional hazards assumption. For the primary analyses, statistical significance was defined as a two-sided *p* value < 0.05. Absolute 3-year event proportions were reported as the number of patients with an outcome divided by the number of eligible patients at risk for that outcome. No formal adjustment for multiple comparisons was applied to the subgroup or stratified analyses, which were considered exploratory and hypothesis-generating. All statistical analyses were performed on the TriNetX platform, which uses R version 4.0.2 with Java version 11.0.16 and Python version 3.7 as its underlying analytical engine. Study size was determined by inclusion of all eligible patients available in the TriNetX US Collaborative Network during the study period; no a priori sample-size calculation was performed.

## 3. Results

### 3.1. Demographic Characteristics of the Study Population

Baseline characteristics are presented in Table 1. After 1:1 PSM, each cohort contained 20,894 patients (Figure 1). Before PSM, the strabismus cohort had a higher mean age than the non-strabismus cohort (71.8 ± 5.2 years vs. 70.3 ± 4.9 years, *p* < 0.001), a lower proportion of women (53.6% vs. 58.0%, *p* < 0.001), a higher proportion of White patients (79.1% vs. 70.2%, *p* < 0.001), and a higher proportion of non-Hispanic patients (80.5% vs. 72.8%, *p* < 0.001). The strabismus cohort also had higher prevalences of cerebrovascular disease, age-related cataract, lacrimal system disorders, and orbital disorders, whereas the comparison cohort had higher prevalences of hypertension, dyslipidemia, and diabetes mellitus. After matching, most baseline characteristics were well balanced. A small residual difference remained in ophthalmology service utilization (SMD = 0.078), while orbital disorders remained imbalanced (SMD = 0.138).

### 3.2. Association Between Strabismus and Glaucoma-Related Outcomes

**Figure 2:**
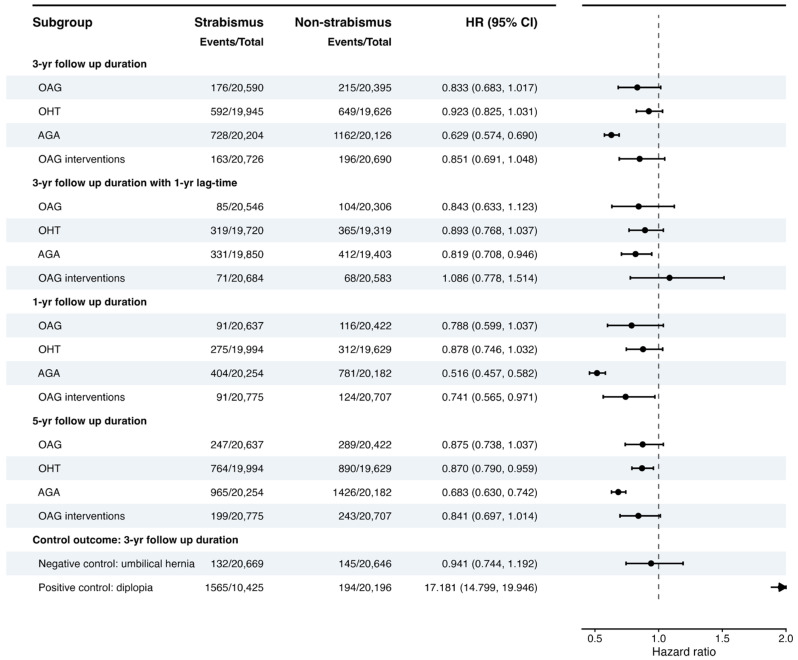
Hazard ratios for OAG, OHT, AGA initiation, and OAG interventions exposed to strabismus compared to non-strabismus. The right-pointing arrow for the positive control outcome indicates that the hazard ratio and its 95% confidence interval exceed the displayed x-axis range.

**Figure 3:**
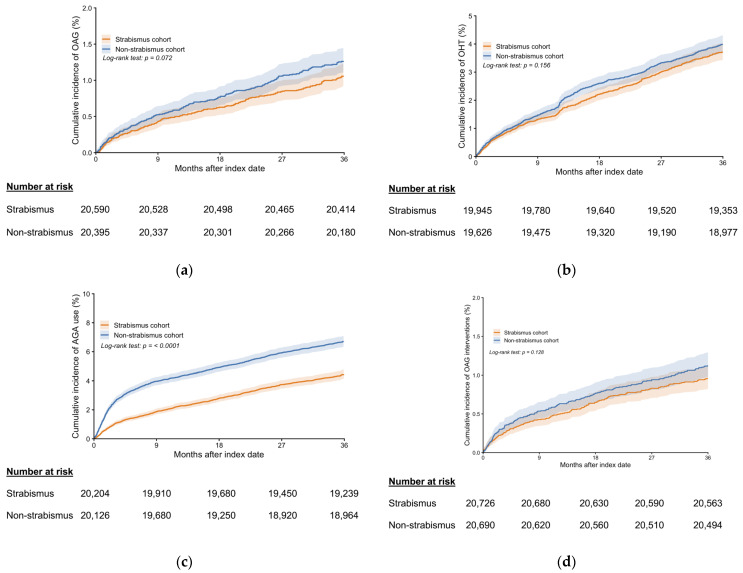
Kaplan–Meier analyses for 3-year follow-up duration risk of OAG and related outcomes. (**a**) OAG, (**b**) OHT, (**c**) AGA initiation, and (**d**) OAG interventions.

HRs for OAG, OHT, AGA initiation, and OAG interventions were assessed over a 3-year follow-up period (Figure 2 and Figure 3).

During the 3-year follow-up, OAG was recorded in 176 of 20,590 patients with strabismus (0.85%) and 215 of 20,395 patients without strabismus (1.05%). The corresponding Kaplan–Meier-estimated 3-year cumulative incidences were 1.06% and 1.27%, respectively, with no significant difference between the survival curves (HR = 0.833 [0.683, 1.017], *p* = 0.072). OHT were recorded in 592 of 19,945 patients with strabismus (2.97%) and 649 of 19,626 patients without strabismus (3.31%), with corresponding cumulative incidences of 3.72% and 3.99% (HR = 0.923 [0.825, 1.031], *p* = 0.156). OAG interventions occurred in 163 of 20,726 patients with strabismus (0.79%) and 196 of 20,690 patients without strabismus (0.95%), with corresponding cumulative incidences of 0.96% and 1.12% (HR = 0.851 [0.691, 1.048], *p* = 0.128).

In contrast, AGA initiation was recorded in 728 of 20,204 patients with strabismus (3.60%) and 1162 of 20,126 patients without strabismus (5.77%). The corresponding cumulative incidences were 4.44% and 6.71%, respectively (HR = 0.629 [0.574, 0.690], *p* < 0.0001). The proportional hazards assumption was not violated for OAG, OHT, or OAG interventions; however, evidence of non-proportional hazards was observed for AGA initiation (*p* < 0.001), and the corresponding HR should therefore be interpreted as an overall summary estimate.

**Figure 4:**
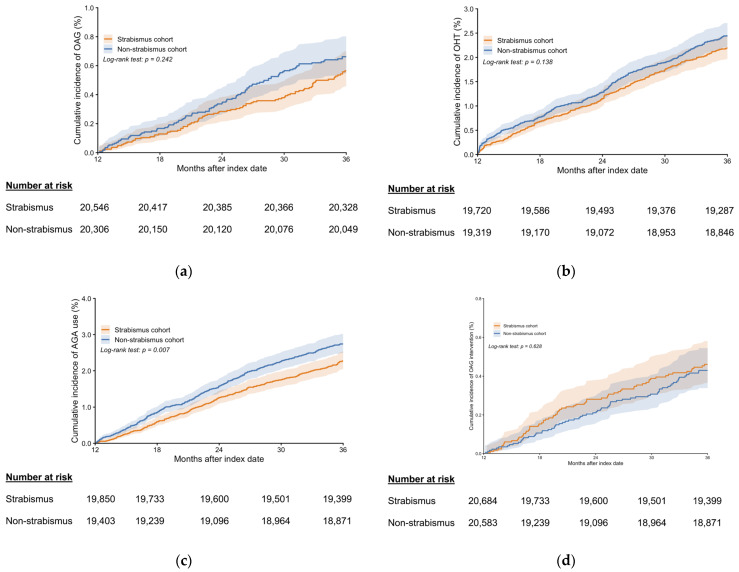
Kaplan–Meier analyses for 3-year follow-up duration with 1-year lag time risk of OAG and related outcomes. (**a**) OAG, (**b**) OHT, (**c**) AGA initiation, and (**d**) OAG interventions.

Findings were similar in sensitivity analyses. In 1-year lag-time analysis, after exclusion of events occurring within the first year after the index date, risks remained nonsignificant for OAG (HR = 0.843 [0.633, 1.123], *p* = 0.242), OHT (HR = 0.893 [0.768, 1.037], *p* = 0.138), and OAG interventions (HR = 1.086 [0.778, 1.514], *p* = 0.628) (Figure 4). Lower AGA initiation persisted, although the effect size was attenuated (HR = 0.819 [0.708, 0.946], *p* = 0.007). Directionally similar results were observed in analyses using 1-year and 5-year follow-up windows (Figures S1 and S2).

For control outcomes, diplopia behaved as expected for a positive control: the strabismus cohort had a markedly higher risk than the matched control cohort (HR = 17.181 [14.799, 19.946], *p* < 0.0001). In contrast, umbilical hernia, selected as a biologically unrelated negative control, was not significantly associated with strabismus (HR = 0.941 [0.744, 1.192], *p* = 0.615) (Figure S3).

### 3.3. Risk of Glaucoma Among Different Subtypes of Strabismus

No subtype showed a significantly increased 3-year risk of OAG, OHT, or glaucoma-related interventions (Table 2; Figure S4). All four subtypes were associated with lower AGA initiation during the primary 3-year analysis, with HRs ranging from 0.465 in paralytic strabismus to 0.832 in exotropia. However, these inverse associations were largely attenuated in the lag-time models. Since several subtype-specific analyses contained few outcome events and wide confidence intervals, these results should be interpreted as exploratory and should not be considered evidence of equivalence between subtypes.

### 3.4. Stratified Analysis Between Patients with and Without Strabismus

In exploratory stratified analyses, most subgroups showed no clear difference in OAG risk between the cohorts (Figure S5), except for a nominally lower OAG risk observed in patients aged 65–74 years (HR = 0.764 [0.612, 0.956], *p* = 0.042) whereas no corresponding association was observed among patients aged ≥75 years (HR = 0.899 [0.670, 1.206], *p* = 0.320).

## 4. Discussion

The association between strabismus and OAG in the elderly population remains contentious and has not been extensively explored through longitudinal methodologies. Our findings suggest that strabismus was not associated with an increased risk of OAG or OHT. Although prior cross-sectional and case–control studies identified strabismus as a potential risk factor [10,17], this association was not observed in our longitudinal analysis. To our knowledge, this is the first large-scale, multicenter cohort study to rigorously evaluate this relationship in patients aged 65 and older.

### 4.1. Novel Findings and Clinical Implications

Our analysis suggests that strabismus was not associated with an increased risk of OAG, OHT, or OAG interventions. These findings remained robust across various temporal analyses. Furthermore, exploratory subgroup analyses did not identify a consistently increased hazard in any specific strabismus subtype; however, the estimates were imprecise because of the limited number of events.

Previous studies have demonstrated that during horizontal eye movements, as adduction reaches approximately 26 degrees, the optic nerve becomes a taut tether that induces globe retraction in patients with OAG, facilitating posterior bowing of the LC and compressing axons that can operate independently of intraocular pressure [9]. The association between POAG prevalence and esodeviation (OR = 7.61) was also reported in cross-sectional studies [10]. Tethering further reduces ONH blood flow [11] and impairs episcleral venous drainage, causing OHT and LC pore remodeling [12,16]. In patients aged over 65, increased scleral stiffness and reduced strain capacity diminish the eye’s ability to dissipate these tractional forces, leading to permanent axonal damage [13,14,15]. However, despite these biologically plausible mechanisms and prior observational findings, our longitudinal cohort study did not demonstrate a significant association between strabismus and OAG-related outcomes.

### 4.2. Discrepancies with Previous Literature and the Possible Impact of Surveillance Bias

The discrepancy between our longitudinal findings and existing literature warrants careful consideration. Our study did not identify an increased recorded risk of OAG or related outcomes among older adults with strabismus, in contrast to previous studies reporting positive associations between these conditions. Previous studies reported positive associations between strabismus and glaucoma, with adjusted odds ratios for POAG ranging from 1.78 in paralytic strabismus to 2.70 in exotropia, and highlighted a strong link to NTG, with risks increasing by over 200% in vertical strabismus [17].

One possible explanation for the discrepancy between our findings and prior studies is differential surveillance and outcome detection, as patients with strabismus may undergo more frequent healthcare encounters.

First, strabismus can substantially impair quality of life. In older adults, it commonly presents with diplopia, blurred vision, and eye strain, symptoms that often lead to repeated ophthalmic evaluations [2]. New-onset strabismus in this population may also prompt extensive investigation to exclude underlying neurologic or systemic disease [3,20]. Management can be prolonged because of recurrence and reoperation, with reported recurrence rates of 20% to 40% for esotropia and 22% to 59% for exotropia [21], while reoperation rates have been reported to increase over time [22], reaching 10.13% within 5 years [23]. Coexisting ocular conditions such as senile cataract may further complicate the clinical course [24]. Cataract extraction has been shown to lower IOP, particularly in eyes with higher baseline IOP [25], and the high rate of postoperative follow-up compliance may further increase opportunities for ongoing ocular surveillance [26]. In addition, binocular diplopia after cataract surgery is a recognized clinical phenomenon that may arise from decompensation of preexisting strabismus or surgical induction [27].

Our baseline data support the possibility of surveillance bias. Before PSM, the strabismus cohort had higher utilization of inpatient services and ophthalmology-specific procedures. Although the control cohort was restricted to patients with at least one ophthalmology encounter and ophthalmology service utilization was included in the propensity-score model, a small residual difference remained after matching (34.9% vs. 31.3%; SMD = 0.078). Although this value was below the prespecified balance threshold of 0.10, it may reflect differences in the frequency, intensity, or type of ophthalmic care that were not fully captured by the available utilization variables. Patients receiving more frequent or specialized ophthalmic evaluations may have greater opportunities for intraocular pressure measurement, optic nerve assessment, glaucoma diagnosis, and antiglaucoma medication initiation. Therefore, differential surveillance and prescribing practices cannot be excluded.

Strabismus in older adults, particularly esotropia, often presents with symptomatic diplopia that triggers high-frequency clinical consultations. One possible explanation for the inverse associations, such as the reduced risk of OHT in esotropia, is differential surveillance and outcome detection. Among all subtypes of strabismus, patients with esotropia tend to present later and with a shorter symptomatic duration than other types of strabismus, causing more severe impairment on visual function [28]. Because new-onset strabismus in the elderly requires intensive diagnostic monitoring to exclude underlying neurogenic etiologies, these patients enter a rigorous “diagnostic net” early in their clinical course [29,30]. This intensive surveillance may facilitate earlier detection and management, such as cataract extraction or treatment of OHT, reducing the likelihood of a subsequent OAG diagnosis [31,32,33]. This interpretation is partly supported by the lag-time analysis. The attenuation of these inverse associations after exclusion of the first year of follow-up suggests that the apparent protective association is less likely to reflect a biological effect and may instead be related to concentrated clinical attention during the initial diagnostic period.

The discordance between lower recorded AGA initiation and the absence of corresponding differences in recorded OAG, OHT, or OAG interventions suggests that medication initiation did not function as a direct surrogate for glaucoma occurrence in this study. The lower rate of recorded AGA initiation warrants cautious interpretation because it was the only statistically significant difference among the four glaucoma-related outcomes and was not accompanied by corresponding differences in OAG, OHT, or OAG interventions. It should not be interpreted as evidence of a biologically protective effect of strabismus. AGA initiation reflects clinical decision-making and may be influenced by IOP, glaucoma severity, prescribing thresholds, treatment preferences, contraindications, and adherence, many of which were unavailable in TriNetX. AGAs may also be used perioperatively to manage transient IOP elevation after cataract surgery or for other indications, including steroid-induced OHT and hypotrichosis [34,35,36,37,38,39,40]. Medication records may incompletely capture dispensing, treatment duration, discontinuation, or prescriptions outside participating organizations. Despite matching, small residual differences remained in ophthalmology services (34.9% vs. 31.3%; SMD = 0.078) and age-related cataract (25.3% vs. 21.9%; SMD = 0.080), suggesting possible differences in surveillance and treatment pathways. The attenuation in the 1-year lag-time analysis and evidence of non-proportional hazards further suggest that early diagnostic, surveillance, prescribing, or documentation patterns may have contributed. Accordingly, this finding should be regarded as hypothesis-generating rather than causal.

### 4.3. Methodological Safeguards Against Surveillance Bias

To mitigate potential surveillance bias, we implemented a multi-faceted framework of methodological safeguards.

The robustness of our longitudinal design was reinforced by a 1-year lag-time analysis. By excluding outcomes occurring within the first year following the index strabismus diagnosis, we minimized protopathic bias—the opportunistic detection of pre-existing, asymptomatic glaucoma during the initial clinical presentation of strabismus.

Furthermore, we ensured the integrity of our incident cohort by applying stringent exclusion criteria. We established a clean baseline by excluding individuals with a prior history of glaucoma-related ICD-10-CM codes, pre-existing use of AGAs, or previous glaucoma-related procedures. This approach was intended to reduce the influence of previously recorded OAG, although undercoding and undocumented pre-existing disease could not be excluded. We also systematically excluded patients with significant anatomical or traumatic confounders (ocular neoplasms, retinal detachment repair, vitrectomy, or eye injuries) to isolate the biomechanical impact of strabismus-related traction from secondary pathological factors.

The internal validity of our findings was bolstered by the utilization of positive and negative controls and the integration of healthcare utilization metrics into our PSM framework. The necessity of controls is underscored by the unique clinical trajectory of strabismus in older adults. Unlike many asymptomatic ocular conditions, strabismus in older adults is often associated with higher healthcare utilization, which may increase opportunities for outcome detection and intervention. Moreover, by balancing the frequency of clinical encounters, we addressed the intensive diagnostic workups that typically drive higher detection rates in symptomatic strabismus patients.

The control analyses supported the internal validity of the overall study design, although residual bias related to healthcare utilization cannot be completely excluded. Collectively, these safeguards were intended to reduce, although not eliminate, surveillance-related bias.

### 4.4. Strengths and Limitations

This study has several strengths. To our knowledge, it is the first multi-institutional, large-scale retrospective cohort study with a 3-year follow-up period specifically focused on the longitudinal association between strabismus and OAG in patients aged 65 years and older. Second, we examined multiple complementary glaucoma-related outcomes, including diagnostic codes, glaucoma-related interventions, and AGA initiation, to assess the consistency of findings across different outcome definitions. Third, the reliability of our results is reinforced by the implementation of a 1-year lag-time analysis, minimizing potential protopathic bias and accounting for the latent period of glaucoma development. Notably, this study provides a granular analysis of strabismus subtypes, allowing for a better understanding of the anatomical and physiological mechanisms.

Despite these strengths, limitations should be acknowledged. First, because this study relied on the TriNetX federated health research network, diagnoses and outcomes were identified using electronic health record data and ICD-10-CM codes, which may be subject to coding errors, undercoding, and misclassification. Although multiple glaucoma-related outcome definitions were examined, some cases may not have been captured accurately. TriNetX captures diagnoses as recorded during clinical care rather than findings obtained through a standardized protocol. No central adjudication of glaucoma diagnoses was available, and diagnostic thresholds, coding intensity, and coding practices may have varied across participating healthcare organizations. This interinstitutional variability may have introduced additional disease misclassification and outcome heterogeneity. Also, the first recorded strabismus diagnosis in TriNetX may not correspond to the true onset of ocular misalignment. Patients with newly developed strabismus could not be distinguished from those with longstanding strabismus that was newly documented after entering a participating healthcare organization or undergoing an ophthalmologic evaluation. Although lag-time analyses were performed, delayed diagnostic coding remains a potential source of temporal misclassification.

Second, the TriNetX database lacks the granular clinical parameters essential for a comprehensive glaucoma assessment, such as IOP, cup-to-disc ratios, and visual field results, limiting our ability to evaluate the severity or progression of the disease [41,42,43,44,45]. The absence of data regarding strabismus severity, the angle of deviation, or the duration of symptoms prior to the index diagnosis precluded an investigation into a potential dose-dependent relationship between the severity of ocular misalignment and glaucoma risk. Residual confounding by these factors therefore cannot be excluded.

Third, although we attempted to mitigate surveillance bias through matching for healthcare utilization, this bias may not have been entirely eliminated. The symptomatic nature of strabismus in older adults necessitates frequent and intensive ophthalmic follow-up. This “diagnostic net” likely facilitates the opportunistic detection of early-stage or borderline glaucomatous changes that might otherwise remain undiagnosed in the asymptomatic general population.

Fourth, because strabismus and glaucoma-related outcomes were identified from recorded ICD-10-CM, CPT, and medication codes, misclassification and under-ascertainment remain possible. Mild or asymptomatic strabismus may not have been coded, and specific strabismus subtypes may have been inaccurately classified. The first recorded strabismus diagnosis in the database may also represent previously undocumented disease rather than true incident strabismus.

Finally, the external validity of our findings is limited by the age-restricted study population. All participants were aged 65 years or older at the index event; therefore, these findings should not be generalized to younger adults or to patients with childhood-onset or longstanding strabismus. These populations may differ in strabismus etiology, disease duration, ocular biomechanics, comorbidity profiles, and patterns of glaucoma surveillance and management. Further studies across broader age groups are warranted.

## 5. Conclusions

This large multicenter retrospective cohort study did not identify an increased recorded risk of OAG, OHT, or OAG interventions among adults aged 65 years or older with first-recorded strabismus. The lower rate of recorded AGA initiation should not be interpreted as evidence of a protective effect and may reflect differences in surveillance, prescribing practices, treatment indications, or medication capture. Because glaucoma screening strategies were not directly evaluated, clinical assessment should remain individualized according to established risk factors and comprehensive ophthalmic findings.

## Figures and Tables

**Table 1:** Demographic characteristics of strabismus and non-strabismus.

		Before PSM ^2^			After PSM		
		Strabismus (*n* = 22,291)	Non-Strabismus (*n* = 365,753)	SMD ^2^	Strabismus (*n* = 20,894)	Non-Strabismus (*n* = 20,894)	SMD
	Age at index, years	71.8 ± 5.2	70.3 ± 4.9	0.300	71.5 ± 5.0	71.2 ± 5.8	0.052
Sex, *n* (%) ^1^	Female	11,902 (53.6)	211,303 (58.0)	0.090	11,224 (53.7)	11,252 (53.9)	0.003
	Male	10,304 (46.4)	152,709 (41.9)	0.090	9668 (46.3)	9635 (46.1)	0.003
Ethnicity, *n* (%)	Hispanic or Latino	966 (4.3)	25,084 (6.9)	0.110	949 (4.5)	957 (4.6)	0.002
	Not Hispanic or Latino	17,885 (80.5)	265,211 (72.8)	0.183	16,715 (80.0)	16,729 (80.1)	0.002
Race, *n* (%)	White	17,573 (79.1)	255,625 (70.2)	0.206	16,417 (78.6)	16,414 (78.6)	<0.001
	Black or African American	1477 (6.7)	49,303 (13.5)	0.230	1455 (7.0)	1389 (6.6)	0.013
	Asian	824 (3.7)	16,923 (4.6)	0.047	792 (3.8)	753 (3.6)	0.010
	American Indian or Alaska Native	72 (0.3)	1307 (0.4)	0.006	67 (0.3)	79 (0.4)	0.010
	Native Hawaiian or Other Pacific Islander	58 (0.3)	1917 (0.5)	0.042	57 (0.3)	58 (0.3)	0.001
	Other race	440 (2.0)	10,049 (2.8)	0.051	432 (2.1)	389 (1.9)	0.015
Diagnoses/Comorbidities, *n* (%)	Family history of glaucoma	88 (0.4)	1042 (0.3)	0.019	82 (0.4)	63 (0.3)	0.015
	Myopia	1737 (7.8)	19,758 (5.4)	0.096	1603 (7.7)	1350 (6.5)	0.047
	Diabetes mellitus (E08–E13)	4318 (19.4)	95,782 (26.3)	0.164	4110 (19.7)	3759 (18.0)	0.043
	Heart failure (I50)	1417 (6.4)	23,222 (6.4)	<0.001	1304 (6.2)	1291 (6.2)	0.003
	Cerebrovascular diseases (I60–I69)	3402 (15.3)	33,214 (9.1)	0.190	2990 (14.3)	3361 (16.1)	0.049
	Chronic kidney disease (N18)	1769 (8.0)	30,955 (8.5)	0.020	1631 (7.8)	1538 (7.4)	0.017
	Overweight and obesity (E66)	2873 (12.9)	59,380 (16.3)	0.096	2739 (13.1)	2618 (12.5)	0.017
	Nicotine dependence (F17)	1409 (6.3)	28,537 (7.8)	0.058	1333 (6.4)	1283 (6.1)	0.010
	Dyslipidemia (E78)	8500 (38.3)	162,269 (44.6)	0.128	7990 (38.2)	7679 (36.8)	0.031
	Age-related cataract (H25)	5788 (26.1)	72,728 (20.0)	0.145	5282 (25.3)	4573 (21.9)	0.080
	Thyroid disorders (E00–E07)	4167 (18.8)	58,256 (16.0)	0.073	3662 (17.5)	3865 (18.5)	0.025
	Sleep disorders (G47)	3922 (17.7)	63,286 (17.4)	0.007	3629 (17.4)	3628 (17.4)	<0.001
	Socioeconomic/psychosocial hazards (Z55–Z65)	446 (2.0)	8354 (2.3)	0.020	420 (2.0)	407 (1.9)	0.004
	Hypermetropia (H52.0)	1197 (5.4)	15,461 (4.2)	0.053	1121 (5.4)	942 (4.5)	0.040
	Degenerative myopia (H44.2)	165 (0.7)	1059 (0.3)	0.063	143 (0.7)	166 (0.8)	0.013
	Liver diseases (K70–K77)	1338 (6.0)	25,157 (6.9)	0.036	1261 (6.0)	1181 (5.7)	0.016
	Astigmatism (H52.2)	2100 (9.5)	22,610 (6.2)	0.121	1911 (9.1)	1639 (7.8)	0.047
	Rheumatoid arthritis with RF (M05)	114 (0.5)	2418 (0.7)	0.020	111 (0.5)	116 (0.6)	0.003
	Other rheumatoid arthritis (M06)	571 (2.6)	9581 (2.6)	0.004	527 (2.5)	521 (2.5)	0.002
	Ankylosing spondylitis (M45)	34 (0.2)	499 (0.1)	0.004	31 (0.1)	31 (0.1)	<0.001
	Other inflammatory spondylopathies (M46)	525 (2.4)	7877 (2.2)	0.013	475 (2.3)	424 (2.0)	0.017
	Hypertensive diseases (I10–I1A)	9475 (42.7)	175,910 (48.3)	0.114	8824 (42.2)	8580 (41.1)	0.024
	Ischemic heart diseases (I20–I25)	3454 (15.6)	56,731 (15.6)	0.001	3193 (15.3)	3143 (15.0)	0.007
	Migraine (G43)	1058 (4.8)	13,147 (3.6)	0.058	964 (4.6)	1025 (4.9)	0.014
	Tobacco use (Z72.0)	453 (2.0)	10,200 (2.8)	0.050	434 (2.1)	425 (2.0)	0.003
	Systemic connective tissue disorders (M30–M36)	789 (3.6)	10,395 (2.9)	0.040	707 (3.4)	655 (3.1)	0.014
	Chronic lower respiratory diseases (J40–J4A)	3308 (14.9)	59,143 (16.2)	0.037	3086 (14.8)	3016 (14.4)	0.009
	Diseases of arteries/arterioles/capillaries (I70–I79)	3032 (13.7)	46,971 (12.9)	0.022	2773 (13.3)	2733 (13.1)	0.006
	Intracranial injury (S06)	635 (2.9)	4960 (1.4)	0.104	548 (2.6)	765 (3.7)	0.060
	Parkinson’s disease (G20)	721 (3.2)	3724 (1.0)	0.154	564 (2.7)	860 (4.1)	0.078
	Vitamin D deficiency (E55)	2509 (11.3)	48,154 (13.2)	0.059	2358 (11.3)	2297 (11.0)	0.009
	Lacrimal system disorders (H04)	2905 (13.1)	31,219 (8.6)	0.145	2492 (11.9)	2342 (11.2)	0.022
	Benign intracranial hypertension (G93.2)	46 (0.2)	378 (0.1)	0.026	40 (0.2)	54 (0.3)	0.014
	Other cataract (H26)	3095 (13.9)	33,678 (9.3)	0.147	2714 (13.0)	2521 (12.1)	0.028
	Multiple sclerosis (G35)	247 (1.1)	1987 (0.5)	0.063	215 (1.0)	301 (1.4)	0.037
	Corneal scars and opacities (H17)	207 (0.9)	2432 (0.7)	0.030	190 (0.9)	165 (0.8)	0.013
	Other disorders of cornea (H18)	740 (3.3)	7056 (1.9)	0.087	633 (3.0)	579 (2.8)	0.015
	Disorders of orbit (H05)	765 (3.4)	1881 (0.5)	0.211	417 (2.0)	923 (4.4)	0.138
	Unspecified kidney failure (N19)	118 (0.5)	2318 (0.6)	0.014	114 (0.5)	105 (0.5)	0.006
	Neoplasms (C00–D49)	7552 (34.0)	127,206 (34.9)	0.020	7048 (33.7)	6838 (32.7)	0.021
Procedures (healthcare utilization), *n* (%)	Hospital inpatient/observation services	3531 (15.9)	44,001 (12.1)	0.110	3199 (15.3)	3403 (16.3)	0.027
	Office/outpatient services	13,056 (58.8)	221,357 (60.8)	0.041	12,082 (57.8)	11,842 (56.7)	0.023
	Ophthalmology services/procedures	8567 (38.6)	69,642 (19.1)	0.439	7300 (34.9)	6535 (31.3)	0.078
Medications, *n* (%)	Corticosteroids, plain (S01BA)	8123 (36.6)	115,530 (31.7)	0.102	7389 (35.4)	7255 (34.7)	0.013
	Corticosteroids for systemic use (H02)	9634 (43.4)	141,462 (38.9)	0.092	8812 (42.2)	8711 (41.7)	0.010
	Beta blocking agents (C07)	5139 (23.1)	68,123 (18.7)	0.109	4625 (22.1)	4520 (21.6)	0.012
	Calcium channel blockers (C08)	6388 (28.8)	95,648 (26.3)	0.056	5859 (28.0)	5778 (27.7)	0.009
	Agents acting on the renin-angiotensin system (C09)	4523 (20.4)	75,166 (20.6)	0.007	4181 (20.0)	4165 (19.9)	0.002

^1^ Data are presented as *n* (%) unless otherwise indicated. An SMD < 0.1 indicates a negligible difference between groups. ^2^ Abbreviations: PSM, propensity score matching; SMD, standardized mean difference.

**Table 2:** Risk of OAG, OHT, AGA initiation, and OAG interventions among different subtypes of strabismus.

Subtypes of Strabismus		Strabismus		Non-Strabismus		HR (95% C.I.)
		*n*	No. of Event	*n*	No. of Event	
3-yr follow-up duration						
Paralytic strabismus (H49)	OAG	7331	62	7205	75	0.858 (0.613, 1.202)
	OHT/preglaucoma	7093	191	6959	232	0.847 (0.700, 1.026)
	AGA initiation	7205	209	7144	454	0.465 (0.395, 0.548)
	OAG interventions	7369	66	7340	78	0.881 (0.634, 1.222)
Esotropia (H50.0)	OAG	5749	46	5646	63	0.728 (0.498, 1.065)
	OHT/preglaucoma	5604	155	5456	200	0.764 (0.620, 0.943)
	AGA initiation	5611	199	5591	312	0.636 (0.533, 0.760)
	OAG interventions	5769	43	5739	55	0.786 (0.528, 1.172)
Exotropia (H50.1)	OAG	4416	39	4344	54	0.731 (0.484, 1.104)
	OHT/preglaucoma	4279	142	4138	139	1.017 (0.805, 1.285)
	AGA initiation	4332	212	4281	257	0.832 (0.693, 0.998)
	OAG interventions	4473	38	4460	37	1.050 (0.668, 1.651)
Vertical strabismus (H50.2)	OAG	5106	50	4981	58	0.866 (0.593, 1.264)
	OHT/preglaucoma	4959	140	4790	167	0.831 (0.664, 1.040)
	AGA initiation	5008	163	4926	293	0.551 (0.455, 0.668)
	OAG interventions	5109	41	5093	51	0.822 (0.545, 1.240)
3-yr follow-up duration with 1-yr lag-time						
Paralytic strabismus (H49)	OAG	7022	28	6895	34	0.863 (0.523, 1.424)
	OHT/preglaucoma	6719	86	6556	107	0.833 (0.628, 1.107)
	AGA initiation	6816	92	6568	132	0.717 (0.549, 0.935)
	OAG interventions	7045	25	7007	37	0.717 (0.432, 1.192)
Esotropia (H50.0)	OAG	5551	19	5460	32	0.603 (0.342, 1.063)
	OHT/preglaucoma	5369	88	5161	92	0.947 (0.707, 1.268)
	AGA initiation	5317	77	5185	100	0.776 (0.576, 1.044)
	OAG interventions	5561	13	5530	20	0.667 (0.332, 1.340)
Exotropia (H50.1)	OAG	4226	19	4170	17	1.138 (0.592, 2.189)
	OHT/preglaucoma	4052	74	3949	72	1.027 (0.743, 1.421)
	AGA initiation	4030	77	3979	86	0.920 (0.676, 1.251)
	OAG interventions	4276	14	4268	18	0.800 (0.398, 1.608)
Vertical strabismus (H50.2)	OAG	5201	28	5051	22	1.291 (0.739, 2.257)
	OHT/preglaucoma	5010	84	4830	83	1.020 (0.753, 1.381)
	AGA initiation	5032	79	4853	89	0.899 (0.664, 1.217)
	OAG interventions	5202	17	5178	20	0.884 (0.463, 1.688)

## Data Availability

The data supporting the findings of this study were obtained from the TriNetX federated health research network. Restrictions apply to the availability of these data, which were used under license for the current study and are not publicly available because of privacy, legal, and ethical restrictions. The aggregated results generated from the TriNetX platform and the coding algorithms used to define the cohorts, outcomes, and control conditions are included in this article and its Supplementary Materials. Further inquiries may be directed to the corresponding author, subject to institutional approval and TriNetX data-use restrictions.

## Supplementary Materials

The following supporting information can be downloaded at: https://www.mdpi.com/article/10.3390/jcm15155823/s1, Table S1: Cohort definition and inclusion/exclusion criteria for the strabismus cohort; Table S2: Cohort definition and inclusion/exclusion criteria for the non-strabismus cohort; Table S3: Operational definitions of study outcomes and control conditions based on ICD-10-CM, CPT, and medication codes; Figure S1: Kaplan–Meier analyses for 1-year follow-up outcomes, including (a) OAG, (b) OHT/preglaucomatous findings, (c) AGA initiation, and (d) OAG interventions; Figure S2: Kaplan–Meier analyses for 5-year follow-up outcomes, including (a) OAG, (b) OHT/preglaucomatous findings, (c) AGA initiation, and (d) OAG interventions; Figure S3: Kaplan–Meier analyses for the positive control outcome, diplopia, and the negative control outcome, umbilical hernia, including (a) diplopia and (b) umbilical hernia; Figure S4: Kaplan–Meier analyses for risk of OAG among different subtypes of strabismus, including (a) paralytic strabismus, (b) esotropia, (c) exotropia, and (d) vertical strabismus; Figure S5: Stratification analysis of risk of OAG, OHT, AGA initiation, and OAG interventions among different groups.

## Abbreviations

The following abbreviations are used in this manuscript:

AGA	Antiglaucoma agent
CI	Confidence interval
CPT	Current Procedural Terminology
EHR	Electronic health record
HCO	Healthcare organization
HR	Hazard ratio
ICD-10-CM	International Classification of Diseases, Tenth Revision, Clinical Modification
IOP	Intraocular pressure
LC	Lamina cribrosa
NTG	Normal-tension glaucoma
OAG	Open-angle glaucoma
OHT	Ocular hypertension
ONH	Optic nerve head
POAG	Primary open-angle glaucoma
PSM	Propensity score matching
SMD	Standardized mean difference
STROBE	Strengthening the Reporting of Observational Studies in Epidemiology
