# Supplementary material for: Association Between Strabismus and Risk of Open-Angle Glaucoma: A Multicenter Retrospective Cohort Study
Source: J Clin Med. 2026 Jul 25;15(15):5823. doi: 10.3390/jcm15155823 (PMC13466239; doi:10.3390/jcm15155823)
Supplement: Supplementary file 1 [file jcm-15-05823-s001.zip › jcm-4415505-supplementary.pdf]

Journal of
Clinical Medicine

MDPI

Article

Association Between Strabismus and Risk of Open-Angle Glaucoma: A Multicenter Retrospective Cohort Study

Chen-Ya Hung [1,†], Shu-Han Chuang [2,†], Bing-Hua Lin [3], Yu-Pin Chen [4,5], Yi-Jie Kuo [4,5], Cheng-Hsien Chang [2,6], Lien-Chen Wu [4,5,7] and Wei-Yang Lu [2,6,8,*]

[1] SSchool of Medicine, College of Medicine, Taipei Medical University, Taipei 11031, Taiwan
[2] Department of Ophthalmology, Changhua Christian Hospital, Changhua 50006, Taiwan
[3] Division of General Practice, Department of Medical Education, Changhua Christian Hospital, Changhua 50006, Taiwan
[4] Department of Orthopedics, Wan Fang Hospital, Taipei Medical University, Taipei 11031, Taiwan
[5] Department of Orthopedics, School of Medicine, College of Medicine, Taipei Medical University, Taipei 11031, Taiwan
[6] Department of Post-Baccalaureate Medicine, College of Medicine, National Chung Hsing University, Taichung 40227, Taiwan
[7] Graduate Institute of Biomedical Materials and Tissue Engineering, College of Biomedical Engineering, Taipei Medical University, Taipei 11031, Taiwan
[8] Department of Optometry, Chung Shan Medical University, Taichung 40242, Taiwan
* Correspondence: wesley1126@gmail.com
† These authors contributed equally to this work.

Supplementary Material

Table of Contents

Table S1. Cohort definition and inclusion/ exclusion criteria for the strabismus cohort.

Table S2. Cohort definition and inclusion/ exclusion criteria for the non-strabismus cohort.

Table S3. Operational definitions of study outcomes and control conditions based on ICD-10-CM, CPT, and medication codes.

Figure S1. Kaplan–Meier analyses for 1-year follow-up outcomes, including (a) OAG; (b) OHT/ pre-glaucomatous findings; (c) AGA initiation; and (d) OAG interventions.

Figure S2. Kaplan–Meier analyses for 5-year follow-up outcomes, including (a) OAG; (b) OHT/ pre-glaucomatous findings; (c) AGA initiation; and (d) OAG interventions.

Figure S3. Kaplan–Meier analyses for the positive control outcome, diplopia, and the negative control outcome, umbilical hernia, including (a)diplopia and (b) umbilical hernia.

Figure S4. Kaplan-Meier analyses for risk of OAG among different subtypes of strabismus, including (a) paralytic strabismus, (b) esotropia, (c) exotropia and (d) vertical strabismus.

Figure S5. Stratification analysis of risk of OAG, OHT, AGA initiation, and OAG interventions among different groups.

Supplemental Tables

Table S1. Cohort definition and inclusion/ exclusion criteria for the strabismus cohort.

Cohort Setting	Definition
Inclusion criteria	Ophthalmology Services and Procedures: UMLS:CPT:1012793 (must be at least 65 years old at event)<br>AND any of the following diagnoses (must be at least 65 years old at event):<br>Paralytic strabismus: UMLS:ICD10CM:H49.<br>Other strabismus: UMLS:ICD10CM:H50.
Time frame	January 1, 2003 – December 31, 2022
Exclusion (on/before index)	Glaucoma: UMLS:ICD10CM:H40-H42.<br>Antiglaucoma Medications: NLM:VA:OP100.<br>Glaucoma Procedures: Goniotomy (CPT:65820), Trabeculotomy ab externo (CPT:65850), Laser trabeculoplasty (CPT:65855).<br>Destruction Procedures on the Iris/Ciliary Body (CPT:1009859), Fistulization of sclera (CPT:1009830), Filtering bleb status (ICD10CM:Z98.83).<br>Transluminal dilation of aqueous outflow canal (CPT:66174, 66175).<br>Aqueous drainage device insertions (CPT:0474T, 0450T, 0449T, 0376T, 0191T, 0671T).<br>Extracapsular cataract removal with aqueous drainage device (CPT:66989, 66991).<br>Aqueous Shunt Procedures (CPT:1013947), Excision Procedures on Iris/Ciliary Body (CPT:1009849).
General exclusion (any time)	Neoplasms: Malignant/Benign neoplasm of eye (ICD10CM:C69, D31), Malignant melanoma of eyelid (C43.1), Carcinoma in situ of eye (D09.2), Benign neoplasm of head/face/neck soft tissue (D21.0).<br>Retinal/Vitreous Procedures: Retinal detachment repair (CPT:67108, 67113), Vitrectomy (CPT:1014238, 67121).<br>Injury & Congenital: Injury of eye/orbit (ICD10CM:S05), Congenital malformations (Q10-Q15), Glaucoma associated with congenital anomalies (ICD9CM:365.4).<br>Secondary Glaucomas: Pigmentary (H40.13), Capsular (H40.14), Primary angle-closure (H40.2), Secondary to trauma (H40.3), Secondary to inflammation (H40.4), Secondary to other eye disorders (H40.5), Secondary to drugs (H40.6), Glaucoma in diseases classified elsewhere (H42), Anatomical narrow angle (H40.03), Steroid responder (H40.04).<br>Inflammatory & Other Disorders: Iridocyclitis (H20), Chorioretinal inflammation (H30), Sarcoid iridocyclitis (D86.83), Various infectious iridocyclitis (A54.32, B02.32, B00.51, A18.5, B58.0), Retinal vasculitis (H35.06), Endophthalmitis (H44.0, H44.1), Vitreous body disorders (H43.89), Retinopathy of prematurity (H35.1).<br>Retinopathy & Vascular: Proliferative diabetic retinopathy (E11.35, E08.35, E09.35, E10.35, E13.35), Retinal neovascularization (H35.05), Exudative AMD (H35.32), Retinal vascular occlusions (H34).

Table S2. Cohort definition and inclusion/ exclusion criteria for the non-strabismus cohort.

Cohort Setting	Definition
Inclusion criteria	Ophthalmology Services and Procedures: UMLS:CPT:1012793 (must be at least 65 years old at event)
Time frame	January 1, 2003 – December 31, 2022
Exclusion (on/before index)	Glaucoma: UMLS:ICD10CM:H40-H42.<br>Antiglaucoma Medications: NLM:VA:OP100.<br>Glaucoma Procedures: Goniotomy (CPT:65820), Trabeculotomy ab externo (CPT:65850), Laser trabeculoplasty (CPT:65855).

	Destruction Procedures on the Iris/Ciliary Body (CPT:1009859), Fistulization of sclera (CPT:1009830), Filtering bleb status (ICD10CM:Z98.83).<br>Transluminal dilation of aqueous outflow canal (CPT:66174, 66175).<br>Aqueous drainage device insertions (CPT:0474T, 0450T, 0449T, 0376T, 0191T, 0671T).<br>Extracapsular cataract removal with aqueous drainage device (CPT:66989, 66991).<br>Aqueous Shunt Procedures (CPT:1013947), Excision Procedures on Iris/Ciliary Body (CPT:1009849).
General exclusion (any time)	Strabismus: Must NOT have Paralytic strabismus (ICD10CM:H49) or Other strabismus (ICD10CM:H50).<br>Neoplasms: Malignant/Benign neoplasm of eye (ICD10CM:C69, D31), Malignant melanoma of eyelid (C43.1), Carcinoma in situ of eye (D09.2), Benign neoplasm of head/face/neck soft tissue (D21.0).<br>Retinal/Vitreous Procedures: Retinal detachment repair (CPT:67108, 67113), Vitrectomy (CPT:1014238, 67121).<br>Injury & Congenital: Injury of eye/orbit (ICD10CM:S05), Congenital malformations (Q10-Q15), Glaucoma associated with congenital anomalies (ICD9CM:365.4).<br>Secondary Glaucomas: Pigmentary (H40.13), Capsular (H40.14), Primary angle-closure (H40.2), Secondary to trauma (H40.3), Secondary to inflammation (H40.4), Secondary to other eye disorders (H40.5), Secondary to drugs (H40.6), Glaucoma in diseases classified elsewhere (H42), Anatomical narrow angle (H40.03), Steroid responder (H40.04).<br>Inflammatory & Other Disorders: Iridocyclitis (H20), Chorioretinal inflammation (H30), Sarcoid iridocyclitis (D86.83), Various infectious iridocyclitis (A54.32, B02.32, B00.51, A18.5, B58.0), Retinal vasculitis (H35.06), Endophthalmitis (H44.0, H44.1), Vitreous body disorders (H43.89), Retinopathy of prematurity (H35.1).<br>Retinopathy & Vascular: Proliferative diabetic retinopathy (E11.35, E08.35, E09.35, E10.35, E13.35), Retinal neovascularization (H35.05), Exudative AMD (H35.32), Retinal vascular occlusions (H34).

Table S3. Operational definitions of study outcomes and control conditions based on ICD-10-CM, CPT, and medication codes.

Outcomes	Definition
OAG	ICD10CM: H40.11, H40.10, H40.12.
OHT	ICD10CM: H40.00, H40.01, H40.02, H40.05.
AGA	NLM:VA:OP100.
OAG intervention	CPT: 66170, 66180, 66710, 66711, 66989, 66991, 0671T, 0191T, 0376T, 0449T, 0450T, 0474T, 65850, 65820, 1009859, 66175, 1013947, 66150, 66172, 66179, 65855, 1020562.
Negative Control	Umbilical hernia (ICD10CM:K42).
Positive Control	Diplopia (ICD10CM:H53.2).

Supplemental Figures

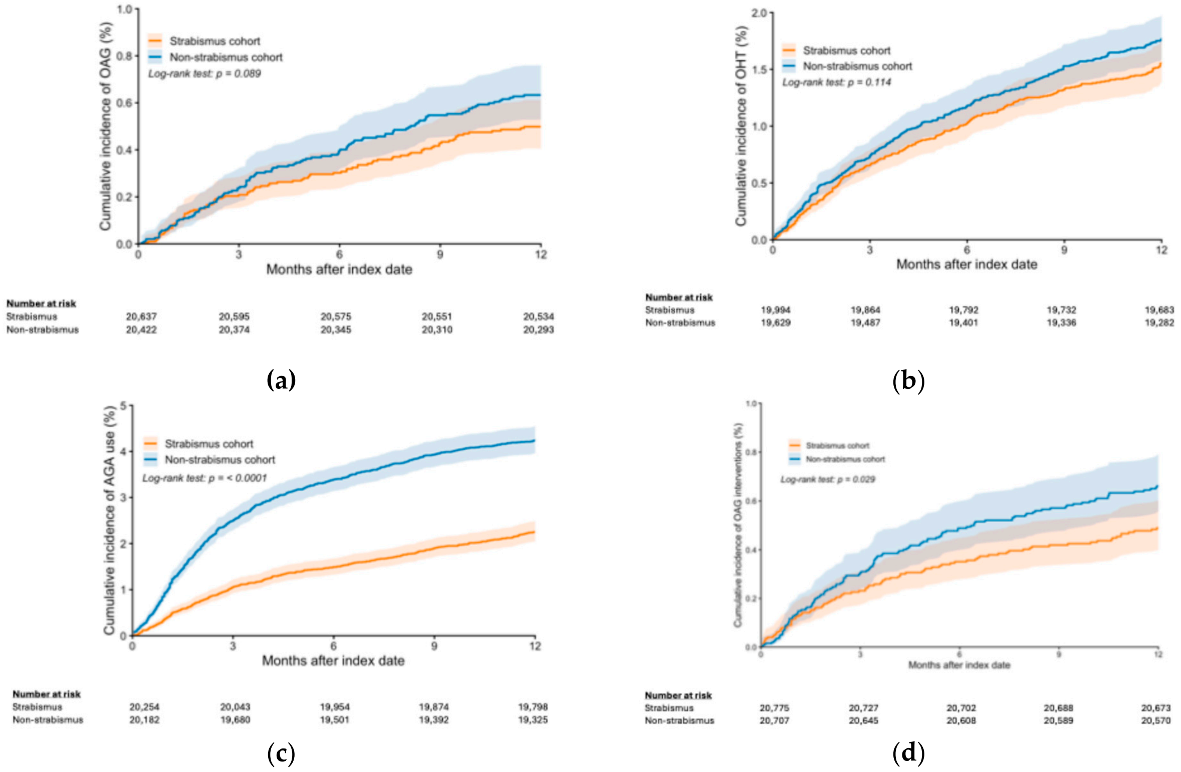

Figure S1. Kaplan–Meier analyses for 1-year follow-up outcomes, including (a) OAG; (b) OHT/ pre-glaucomatous findings; (c) AGA initiation; and (d) OAG interventions.

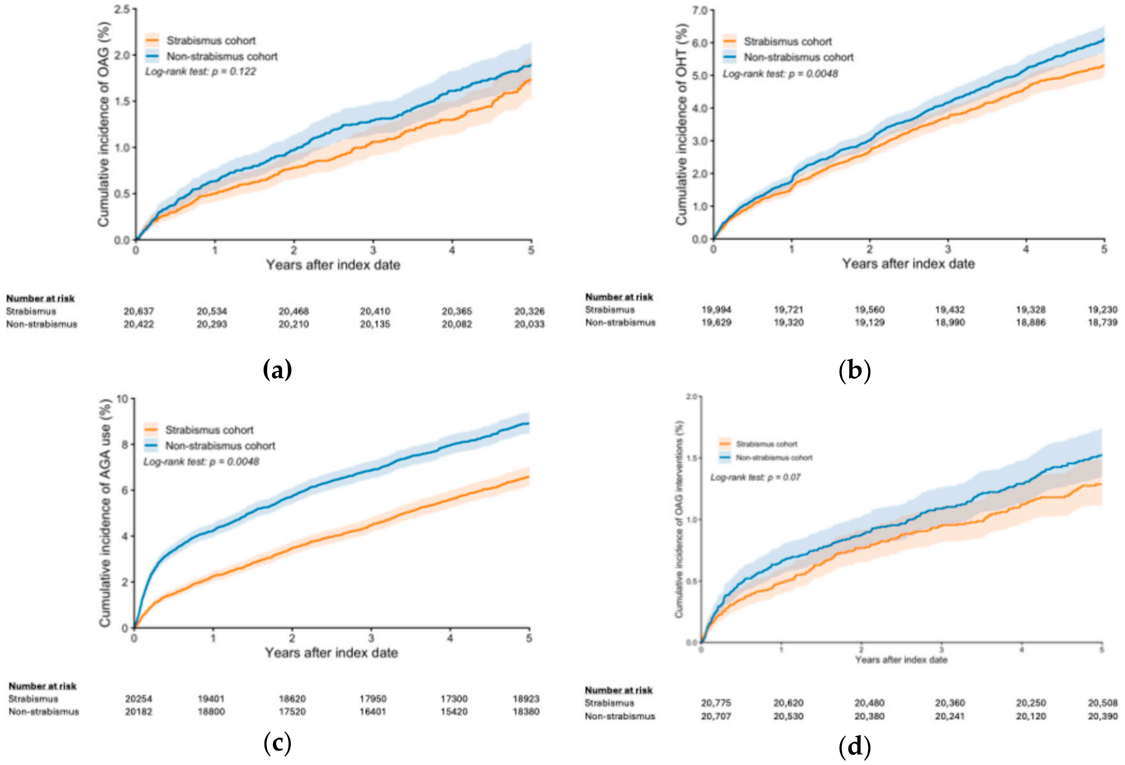

Figure S2. Kaplan–Meier analyses for 5-year follow-up outcomes, including (a) OAG; (b) OHT/ pre-glaucomatous findings; (c) AGA initiation; and (d) OAG interventions.

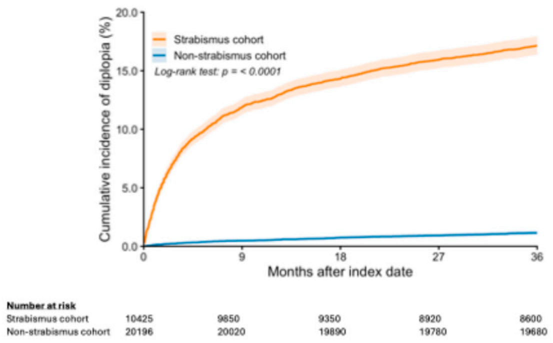

(a)

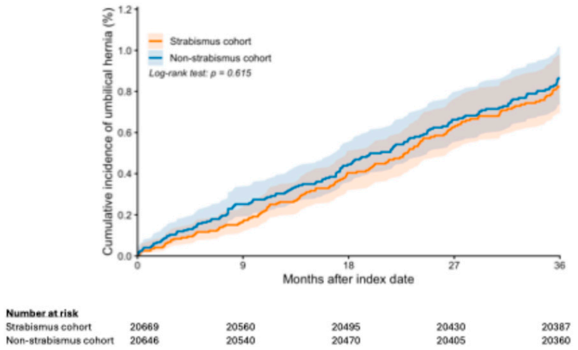

(**b**)

Figure S3. Kaplan–Meier analyses for the positive control outcome, diplopia, and the negative control outcome, umbilical hernia, including (a)diplopia; (b) umbilical hernia.

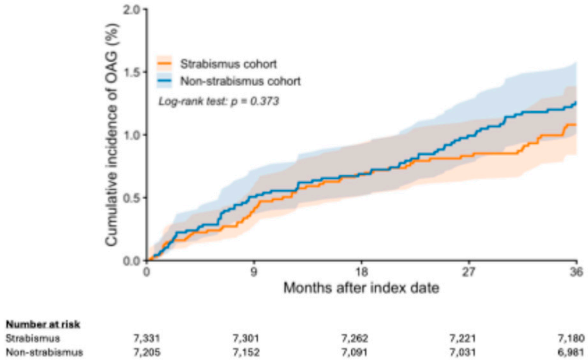

(**a**)

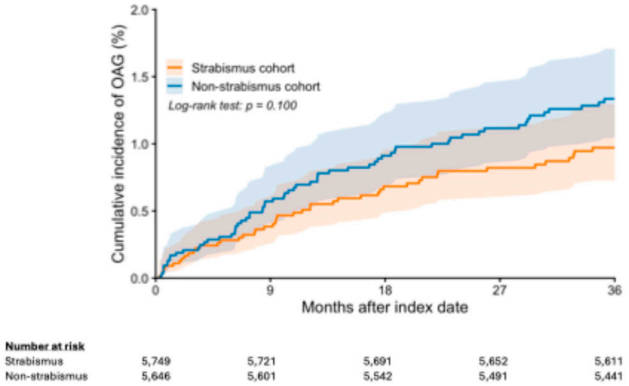

(**b**)

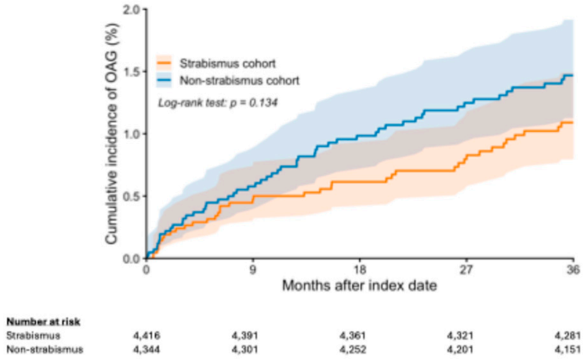

(**c**)

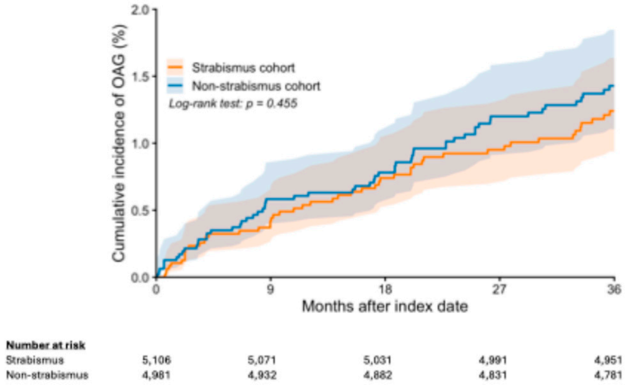

(**d**)

Figure S4. Kaplan-Meier analyses for risk of OAG among different subtypes of strabismus, including (a) paralytic strabismus; (b) esotropia; (c) exotropia; (d) vertical strabismus.

Subgroup	Strabismus No. of event/ N	Non-Strabismus No. of event/ N	HR (95% C.I.)
**Age**			
65-74	135/15,125	180/14,937	0.764 (0.612, 0.956)
≥ 75	84/ 7,635	95/ 7,522	0.899 (0.670, 1.206)
**Sex**			
Female	130/13,129	137/13,045	0.971 (0.764, 1.234)
Male	98/11,445	131/11,342	0.756 (0.582, 0.982)
**Ethnic**			
Hispanic/ latino	16/ 967	13/ 950	1.278 (0.615, 2.657)
Not hispanic/ latino	154/17,687	199/17,471	0.786 (0.637, 0.970)
**Race**			
White	167/19,957	172/19,815	0.986 (0.797, 1.220)
Black or African American	24/ 1,410	29/ 1,404	0.827 (0.481, 1.420)
Asian	13/ 779	14/ 770	0.918 (0.431, 1.952)
**Comorbidity**			
Hypertension	144/14,837	163/14,712	0.891 (0.712, 1.114)
Hyperlipidemia	138/13,858	165/13,762	0.841 (0.671, 1.054)
Diabetes mellitus	75/ 6,967	91/ 6,918	0.820 (0.604, 1.113)
Overweight and obesity	50/ 5,634	75/ 5,591	0.665 (0.465, 0.952)
Nicotine dependence	16/ 2,251	15/ 2,228	1.069 (0.529, 2.162)
Ischemic heart diseases	46/ 6,641	67/ 6,611	0.688 (0.473, 1.001)
Sleep disorders	79/ 7,307	77/ 7,293	1.030 (0.753, 1.410)
Chronic lower respiratory diseases	53/ 5,967	63/ 5,927	0.847 (0.588, 1.221)
Age-related cataract	152/12,537	160/12,452	0.939 (0.752, 1.173)

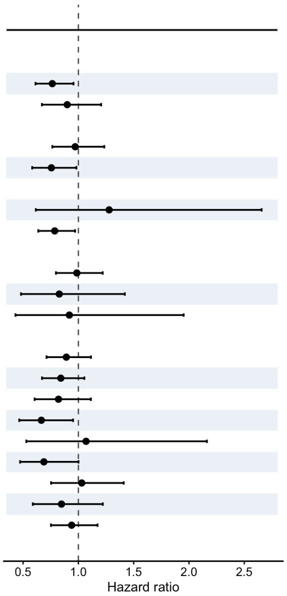

Figure S5: Stratification analysis of risk of OAG, OHT, AGA initiation, and OAG interventions among different groups.
